# Finite Size‐Effects in Martensite Microstructure of Magnetic Shape Memory Films

**DOI:** 10.1002/smll.202512162

**Published:** 2026-02-04

**Authors:** Satyakam Kar, Aman Singh, Kornelius Nielsch, Heiko Reith, Sebastian Fähler

**Affiliations:** ^1^ Institute for Metallic Materials Leibniz IFW Dresden Dresden Germany; ^2^ Institute of Materials Science and Institute of Applied Physics TU Dresden Dresden Germany; ^3^ Helmholtz‐Zentrum Dresden‐Rossendorf Dresden Germany; ^4^ Institute for Emerging Electronic Technologies Leibniz IFW Dresden Dresden Germany

**Keywords:** epitaxial films, magnetic shape memory alloys, martensite microstructure, microfabrication, size‐effect

## Abstract

Magnetic shape memory alloys, owing to their multifunctional properties, are a promising material system for integration into microsystems. Their multifunctionality arises from the coexistence of ferroelasticity and ferromagnetism. While size‐effects in ferromagnetic microstructure are well understood, corresponding experiments on the ferroelastic martensite microstructure are sparse. In this study, we use epitaxially grown Ni‐Mn‐Ga‐based films as a model system to investigate the influence of finite size on the martensite microstructure under constrained and freestanding conditions. The results show that the microfabricated patterns, in both conditions, retain the characteristics of their continuous film microstructures. Film thickness has a strong influence, as this is the smallest extension investigated in our study. Our analysis reveals similarities and differences between finite size effects in ferromagnetic and ferroelastic microstructure, which is crucial for using these multifunctional materials in microsystems.

## Graphical abstract

Magnetic shape memory alloys are a promising multifunctional material system for integration into microsystems. Their multifunctionality arises from the coexistence of ferroelasticity and ferromagnetism. While size‐effects in ferromagnetic microstructure are well understood, corresponding experiments on the ferroelastic microstructure are sparse. Read this article to understand how finite size‐effects appear in the ferroelastic microstructure and differ under constrained and freestanding conditions.

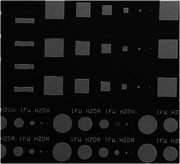

## 1 Introduction

Ni‐Mn‐based magnetic shape memory alloys are multiferroic materials and exhibit reversible ferroelastic and ferromagnetic transitions [1]. Coupling between both ferroic properties unlocks multifunctional effects in these materials, such as magnetic field‐induced actuation [2], reaching a strain up to 12% [3], and actuation frequency up to 100 kHz [4], caloric effects for solid‐state cooling [5, 6], and thermomagnetic harvesting of low‐grade waste heat [7, 8]. This multifunctionality makes magnetic shape memory alloy films a promising candidate for integration into microsystems. However, the success of this integration relies on understanding the influence of miniaturization on the ferroic microstructure of these alloys, critical for their multifunctional effects.

The size‐effects in a ferromagnetic microstructure have been extensively studied [9, 10], and among the multitude of effects, of particular interest for this work are: (1) Edge‐effects, where the magnetic domain arrangement is affected close to the free surfaces created by miniaturization, (2) Single domain particles, and (3) Scaling effects, where the domain width scales with film thickness. These three effects originate from the minimization of total magnetic energy, with competing contributions from magnetostatic energy as the volume energy, and domain wall energy as the interface energy. Analogous to ferromagnetic transition, the ferroelastic transition results in ferroelastic domains connected by twin boundaries [11]. The relevant volume and interface energy terms in the ferroelastic martensite microstructure are elastic energy and twin boundary energy. Despite these analogies, differences exist between ferromagnetism and ferroelasticity. The ferromagnetic transition is second‐order, whereas the ferroelastic transition is first‐order and proceeds by nucleation and growth. Ferromagnetic microstructure distinguishes between two possible directions for a magnetization easy‐axis, while ferroelastic microstructure considers the orientation of the martensite crystal within a ferroelastic domain. Moreover, magnetocrystalline anisotropy aligns magnetization to certain crystal orientations, whereas in martensite twin boundaries are anisotropic as they strongly prefer well‐defined crystallographic orientations [12]. With these similarities and differences, it remains an open question whether the three highlighted size‐effects in ferromagnetic microstructure have counterparts within the ferroelastic martensite microstructure.

In Ni‐Mn‐Ga‐based magnetic shape memory alloys, the ferromagnetic microstructure is strongly coupled to the ferroelastic microstructure by magnetocrystalline anisotropy [13, 14, 15]. Accordingly, minimizing the energy contribution of the ferroelastic subsystem dominates, with the ferromagnetic microstructure adapting to the underlying martensite microstructure. Hence, finite size effects in ferroelastic martensite microstructure must be examined first in these alloys. The martensite microstructure in these alloys is hierarchical, containing twins‐within twins [16, 17, 18]. Using epitaxial films, five nested twin boundary levels, spanning from nanoscale over the mesoscale up to macroscale, have been identified [18]. Finite size‐effects may therefore become relevant at these three length scales.

Up to now, the size‐effect studies in polycrystalline samples of magnetic shape memory alloys like microwires [19, 20], ribbons [21], and foams [22] have shown improved actuation properties as the grain size approaches at least one of the sample extensions, indicating that free surfaces are beneficial. Furthermore, improvements in functional fatigue resistance during superelastic cycling have been demonstrated in micropillars prepared from bulk single crystal [23] and epitaxial film [24]. While size‐effects on the mechanical properties look promising and remain a subject of ongoing research, the size‐effects on the hierarchical martensite microstructure are gradually drawing attention. Takhsha Ghahfarokhi et al. [25] investigated microfabricated patterns with lateral dimensions ranging from 100 to 3 µm from a 200 nm thick epitaxial Ni‐Mn‐Ga film and reported the possibility of engineering martensite microstructure based on pattern orientation. They also reported a decrease in macroscopic boundary density with a reduction in the size of microdisk patterns below 12 µm. However, these findings remain to be verified by a statistical analysis of several patterned structures. In addition, the size‐effects in freestanding Ni‐Mn‐Ga‐based films and patterns are unexplored so far due to complexities in their fabrication and handling. Recently, we demonstrated a silicon microtechnology‐based process flow to fabricate freestanding epitaxial Ni‐Mn‐Ga‐based film and investigate its martensite microstructure [15, 26, 27]. Our observations revealed a fundamentally different microstructure guided by finite size‐effect in the martensitic transformation. Hence, further size‐effects in freestanding films and patterns are expected by modifying film thickness and pattern characteristics.

In this work, we investigate size‐effects in the ferroelastic martensite microstructure of epitaxially grown Ni‐Mn‐Ga‐based films, inspired by the three size‐effects in ferromagnetism listed earlier in the section. We stick to 500 nm thick films for probing the edge effects and single feature orientation patterns. For studying scaling effects with film thickness, we use films with a thickness as low as 50 nm. This manuscript is arranged as follows: we motivate each size‐effect as known from ferromagnetism, followed by the findings in the martensite microstructure observed under constrained and freestanding conditions. We discuss how the martensite microstructure responds to miniaturization to gain deeper insights into the ferroelastic transition and its microstructural features. Our approach is experimental, and consequently, our explanations remain at the phenomenological level. Thus, our work is complementary to more theoretical works that covered particular aspects in depth, such as the scale bridging microstructure [18], nucleation [28], and branching of martensite microstructure [29].

## 2 Results and Discussion

### 2.1 Edge Effects

Edge effects in a ferromagnetic microstructure can appear in two ways. First, the domain boundaries tend to strike orthogonal to a sample edge to minimize the length of the boundary and their associated excess energy. Second, in ferromagnetic materials with strong uniaxial anisotropy, the domain boundaries tend to branch when approaching the sample edge to minimize the magnetostatic energy at the cost of an increased domain boundary length [10].

**FIGURE 1:**
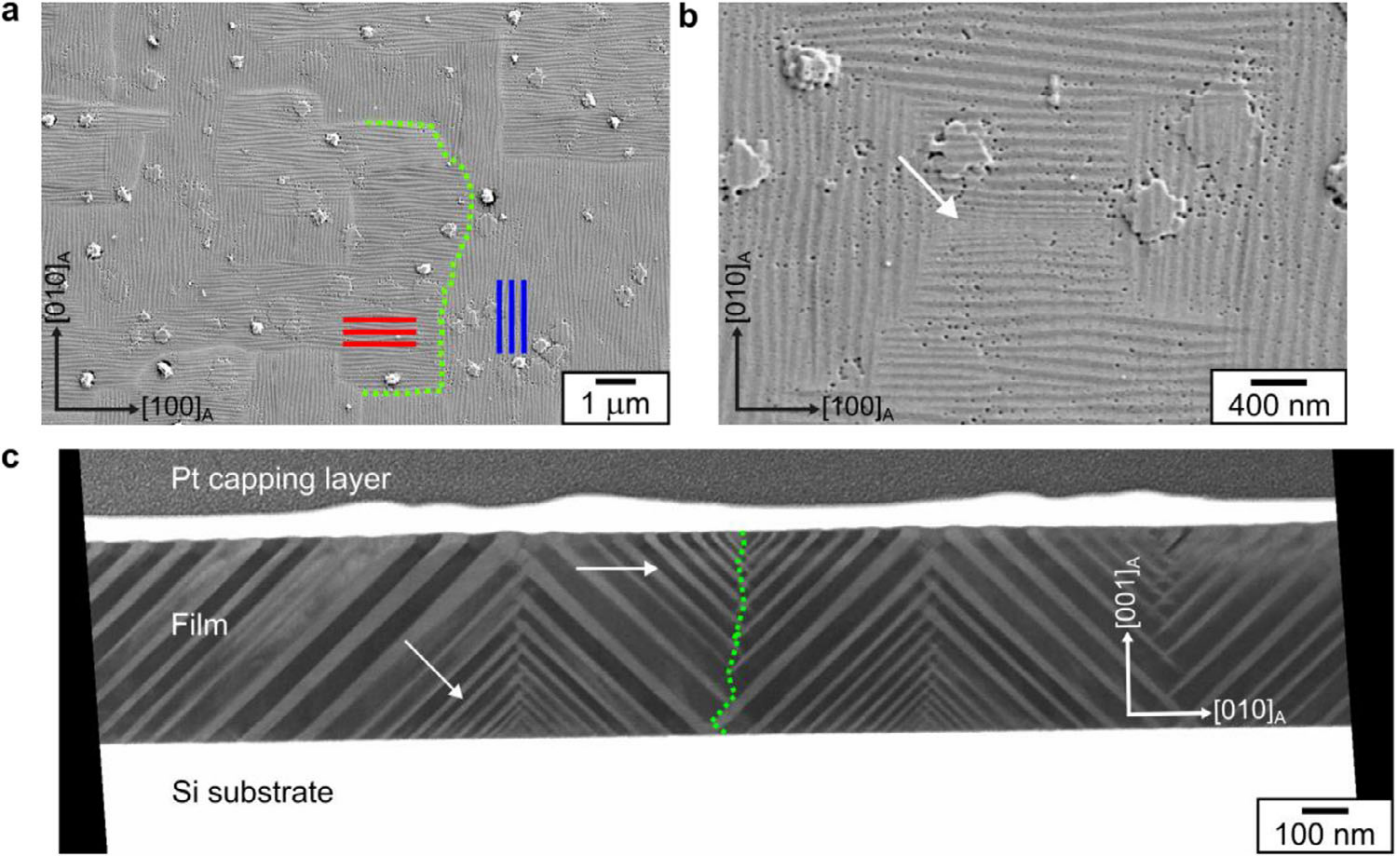
Martensite microstructure of a 500 nm thick Ni_52_Mn_19_Ga_25_Cu_4_ continuous film constrained by the substrate. (a) Secondary electron image of the film surface reveals the hierarchical martensite microstructure. A macroscopic boundary is highlighted by a green dotted line, which separates regions with different mesoscopic twin boundary traces indicated by red and blue lines. (b) A closer look at the martensite microstructure near the arrow reveals a gradually increasing periodicity of mesoscopic twin boundaries. (c) STEM image of the film cross‐section allows to visualize this refinement of twin boundary periodicity at a conjugated macroscopic boundary (marked in green) and close to the substrate, indicated by arrows.

In the martensite microstructure of Ni‐Mn‐Ga films, refinement in the twin boundary period is often noticed at incompatible surfaces and interfaces [18, 30]. Figure 1 depicts the martensite microstructure of a 500 nm thick epitaxial Ni_52_Mn_19_Ga_25_Cu_4_ film grown on a 4 nm SrTiO_3_ buffered Si substrate. The characteristic type X microstructure is observed in the film with two orthogonal traces of mesoscopic twin boundaries [30], highlighted in red and blue (Figure 1a). These boundaries have a period of 108 ± 2 nm. Due to the cubic symmetry of the parent austenite phase, equivalent orientations of mesoscopic twin boundaries can nucleate and grow simultaneously during martensitic transformation [18]. As a result, macroscopic boundaries form between regions differing in mesoscopic twin boundary orientation. The macroscopic boundaries can be considered conjugated or non‐conjugated, depending on similarity or dissimilarity between the traces of mesoscopic twin boundaries on the surface [31]. A non‐conjugated macroscopic boundary is indicated by a green dotted line in Figure 1a. The macroscopic boundaries and the substrate are incompatible interfaces for the growth of mesoscopic twin boundaries. Hence, the twin boundary period reduces close to these interfaces. Such a refinement in the twin boundary period is highlighted in Figure 1b across a conjugated macroscopic boundary. This phenomenon is better visualized in an exemplary cross‐section TEM image of the martensite microstructure (Figure 1c), where the refinement is indicated by arrows.

**FIGURE 2:**
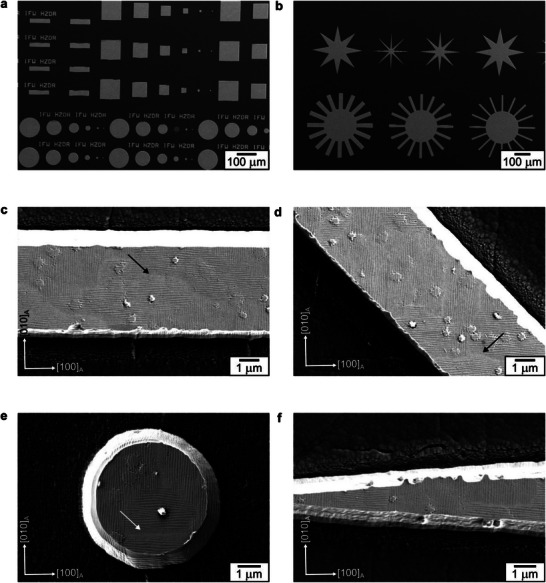
Martensite microstructure in microfabricated Ni_52_Mn_19_Ga_25_Cu_4_ patterns. (a,b) Secondary electron images depict the fabricated patterns, which vary in shape, size, orientation, and aspect ratio. Shown in detail are some of the smallest fabricated patterns: 4 µm wide strips oriented at (c) 0° and (d) 45°, (e) 5 µm disk, and (f) tapering leg of a star‐shaped pattern approaching the sub‐micrometer dimensions. Pattern orientation and aspect ratio do not influence the twin boundary orientation. Local refinement in twin boundary periodicity, similar to Figure 1b, is observed within the patterns, indicated by arrows.

To investigate edge effects in the martensite microstructure, we used the 500 nm‐thick Ni_52_Mn_19_Ga_25_Cu_4_ film and fabricated patterns of different shapes, sizes, and orientations using photolithography and Ar ion‐beam etching process, developed in a previous work [26]. Figure 2a,b gives an overview of some of these patterns, and Figure 2c–f offers a closer look at the martensite microstructure of some of the smallest microfabricated patterns. On analyzing the microstructure in the rectangular stripes (Figure 2c,d), it is evident that the twin boundaries do not exhibit a preferential orientation based on pattern orientation. Both the twin boundary traces (highlighted in Figure 1a) can be identified in these images. The instances of local refinement in the twin boundary period are also observed inside these patterns, highlighted by arrows. These observations also hold for the circular disk and star‐shaped patterns shown in Figure 2e,f, and for larger‐sized patterns not shown here. No refinement is observed close to the pattern edges, which we exemplify using a tapering leg of a star pattern in Figure 2f. These observations show that edge effects do not appear in constrained martensite microstructure upon size reduction and indicate that, in contrast to ferromagnetic domains ferroelastic twin boundaries cannot change their orientation easily.

**FIGURE 3:**
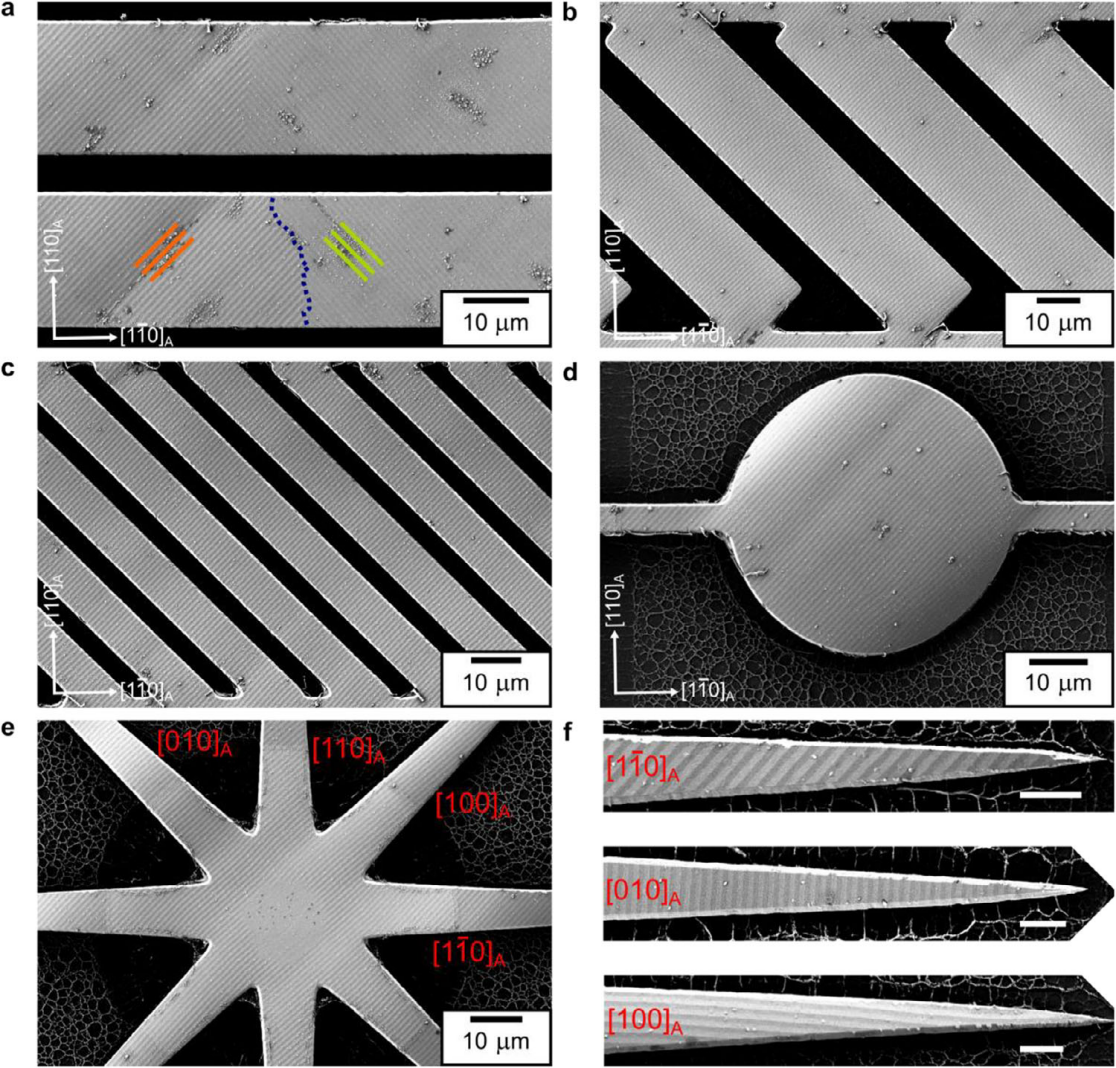
Martensite microstructure in 500 nm thick freestanding Ni_52_Mn_19_Ga_25_Cu_4_ patterns. (a) Secondary electron image of a 20 µm wide freestanding bridge exhibits a parallel stripe microstructure, with two orthogonal stripe orientations marked in orange and green and separated by macroscopic boundary. Array of several freestanding bridges oriented at 45° and width of (b) 20 µm and (c) 8 µm show a single orientation of stripe microstructure. (d) 50 µm freestanding disk and (e) partly freestanding star pattern show a single orientation of stripes with no influence of pattern orientation. (f) Three freestanding legs of the star demonstrating a constant stripe period and orientation as the pattern size reduces. The white bars denote 4 µm. All the patterns have a stripe period of around 1000 nm.

To investigate edge effects in freestanding martensite microstructure, we used a 500 nm thick Ni_52_Mn_19_Ga_25_Cu_4_ film grown on a SrTiO_3_ buffered SOI substrate and etched the film similar to constrained patterns, followed by underetching the top Si layer of SOI substrate using XeF_2_ gas [26]. Figure 3a depicts the martensite microstructure observed in a 20 µm wide freestanding bridge. The microstructure is characterized by a parallel stripe pattern with a period of around 1000 nm. Two orthogonal orientations of stripes occur separated by a macroscopic boundary (indicated by a blue dotted line), as these orientations are crystallographically equivalent. As analyzed in detail in a previous work [15], this microstructure originates from an invariant line constraint during martensitic transformation. Figure 3b,c shows 20 and 8 µm wide freestanding bridges (100 µm long each) oriented along [010]_A_ direction. The stripe period is constant, with no influence of pattern aspect ratio on the orientation of stripes (see Figure S1 for 4 µm wide freestanding bridges). Figure 3d shows a 50 µm wide freestanding disk, which has a single stripe orientation and a stripe period of around 1000 nm. The period and orientation of stripes remain intact close to the pattern edges. We also investigated the microstructure of the partly freestanding star pattern shown in Figure 3e. The pattern is anchored to the substrate at its center and has freestanding legs tapering outward at 45° intervals between 0° and 360°. Their corresponding crystallographic directions are indicated in red. The martensite microstructure on the freestanding legs exhibits a single orientation of stripes along [100]_A_, irrespective of the leg orientation. Notably, the stripe period remains unaffected as the legs taper, which is demonstrated in Figure 3f using legs along [1$\bar{1}$0]_A_, [010]_A,_ and [100]_A_ directions. In the case of [100]_A_ leg, the number of stripes reduces as the leg tapers, with only one stripe extending to the tip. These observations indicate that the stripe microstructure tends to follow a consistent stripe orientation and period, irrespective of pattern orientation and aspect ratio.

To investigate the influence of martensitic transformation in freestanding conditions, we examined the surface microstructure of a partly freestanding star in situ during a heating‐cooling cycle. As shown in Video S1, the martensite microstructure with a single orientation of stripes begins to disappear upon heating to 395 K as the pattern transforms to austenite phase. On cooling, the stripes gradually reappear at 383 K, and the observed region completely transforms to martensite phase at 380 K. The microstructure retains the stripe orientation and periodicity as observed in the as‐released state. These observations suggest that the absence of edge‐effects in the martensite microstructure in freestanding state is not a question of sample history – It does not matter if patterning or the martensitic transformation occurs first.

We do not observe bending of twin boundaries at edges, which occurs for ferromagnetic domain boundaries to minimize their length. The reason for this difference is that twin boundaries must be aligned along a few well‐defined crystallographic planes [30]. Following the phenomenological theory of martensite [32], at the continuum length scale, only these orientations are stress‐free – a constraint which does not exist for magnetic domain walls. As a consequence, the twin boundaries can hardly adapt their orientation to pattern edges. For constrained films, mesoscopic type I twin boundaries follow {110}_A_, while type II twin boundaries deviate a few degrees from this orientation [33]. For freestanding films, the orientation of stripes is constrained along either [010]_A_ or [100]_A_, and their period is consistent to fulfill the invariant line constraint [15].

At the free surface of patterns, the twin boundary period in martensitic microstructure does not exhibit any refinement, unlike branching in a ferromagnetic microstructure. In ferromagnetic materials, a free surface can contribute to magnetic stray field, which increases magnetostatic energy, and can be partly reduced by branching. However, for a ferroelastic material, the free surface does not hinder it from shrinking, expanding, and bending. No additional elastic energy is incurred, and consequently, a reduction in twin boundary period does not occur at pattern edges. It is only observed at incompatible interfaces that pose as a rigid constraint (such as the substrate, macroscopic boundaries, and grain boundaries).

### 2.2 Single Feature Orientation Patterns

Single domain particles in ferromagnetic materials occur when the size of the particles is reduced below a critical size at which the total excess of domain boundary energy, scaling with particle area, cannot anymore compensate the magnetostatic energy, scaling with particle volume [10]. A similar effect is expected for ferroelastic materials, where the twin boundary energy takes the role of surface energy, and elastic energy the role of volume energy. Indeed, single variants of compound twinned martensite had been reported in nanocrystalline NiTi below 100 nm grain size [34]. In the present context, we examine if microfabrication allows to prepare constrained Ni‐Mn‐Ga‐based patterns that contain no macroscopic boundary, as it hinders the movement of highly mobile mesoscopic twin boundary [35].

To ascertain the size limit for our films, we begin by analyzing the martensite microstructure of the continuous 500 nm thick Ni_52_Mn_19_Ga_25_Cu_4_ film as a reference. We use the term ‘mesoscopic twin colony’ to refer to a region containing a single trace orientation of mesoscopic twin boundary. To simplify our analysis, we neglect the macroscopic boundaries that can occur between two mesoscopic twin orientations having the same trace orientation on the film surface. Our microstructure analysis (see Figure S2) reveals several small (<25 µm^2^) and medium‐sized colonies (25–75 µm^2^) among a few large‐sized colonies (>75 µm^2^). Using a 4 µm × 4 µm square grid segmentation of the microstructure images, we observe that out of 120 squares analyzed, 50% have two mesoscopic twin colonies, 37% contain three colonies, and about 11% contain one colony (see Figure S2e). This analysis suggests that the selected square size is close to the limit for the transition from two colonies to one colony per square. Hence, we fabricated an array of 4 µm squares to investigate for size‐effects.

**FIGURE 4:**
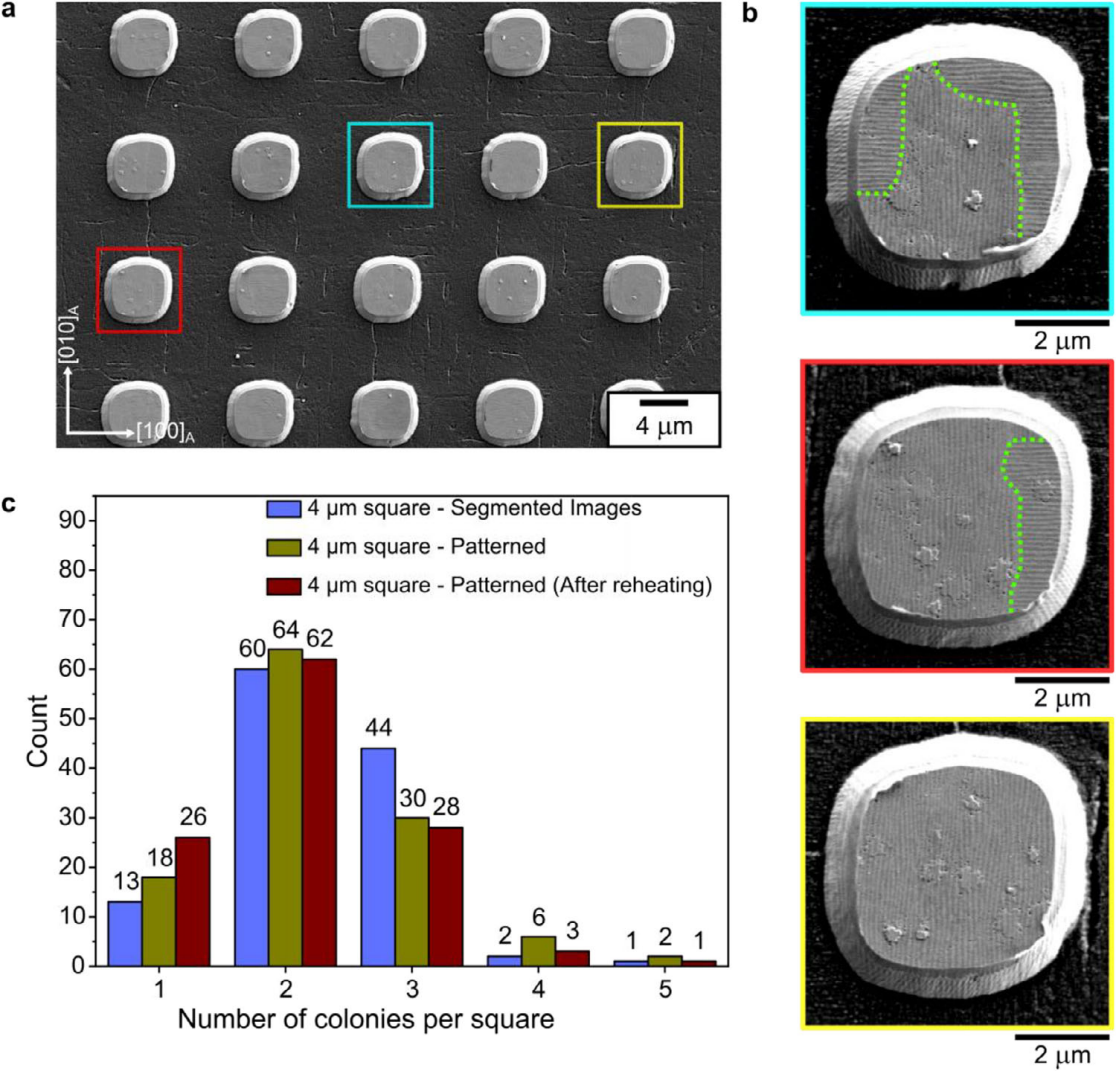
Microstructure analysis of 4 µm microfabricated squares on a 500 nm thick constrained Ni_52_Mn_19_Ga_25_Cu_4_ film. (a) Secondary electron image showing a region of the fabricated array of squares. Three squares, highlighted by blue, red, and yellow boxes, are used to demonstrate the martensite microstructure in (b). Close‐up images of the three highlighted squares exhibit three (blue), two (red), and one (yellow) mesoscopic twin colonies. (c) Bar chart summarizing the colony distribution in 120 squares obtained in three ways: Blue bars – segmented continuous film images as reference state, Green bars – as‐patterned state, and Maroon bars – after a martensitic transformation cycle of the patterns.

Figure 4a exemplarily shows some 4 µm squares obtained through patterning. The period of mesoscopic twin boundaries observed here is 105 ± 4 nm, which is close to that of the continuous film (108 ± 2 nm). It shows that this length scale of the martensitic microstructure is not affected by patterning. The martensite microstructure observed at a larger length scale is similar to the continuous film, as illustrated for three squares (highlighted by boxes) in Figure 4b. These squares have three (blue box), two (red box), and one (yellow box) mesoscopic twin colonies. We analyzed 120 patterned squares to get a representative distribution of mesoscopic twin colonies per square. The results are plotted as green bars in Figure 4c. It shows that the majority of the squares (53%) have two mesoscopic twin colonies, followed by squares with three colonies (25%) and one colony (15%). This trend is identical to that obtained for segmented continuous film images (blue bars), which suggests that the free surface and lateral confinement induced by patterning do not influence the existing martensite microstructure in the constrained state.

Subsequently, we heated the patterned sample to 410 K to achieve the austenite phase and undergo martensitic transformation in the patterned state while cooling to room temperature. This procedure allows to investigate if the pattern edges would influence nucleation and the resulting microstructure. The analyzed microstructure of 120 squares is plotted as maroon bars in Figure 4c. The distribution follows a trend similar to the one obtained after patterning, illustrating that martensitic transformation in the patterned state does not produce a distinct change in the mesoscopic twin colony statistics.

**FIGURE 5:**
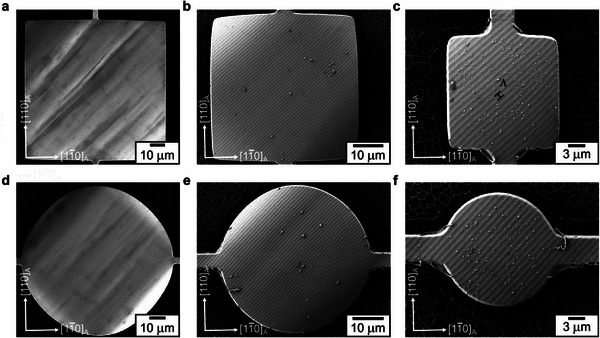
Effect of pattern size on freestanding martensite microstructure. Secondary electron images of square and circular disk‐shaped freestanding patterns fabricated in three sets of sizes (width/diameter) (a,d) 100 µm, (b,e) 50 µm, and (c,f) 20 µm. A single stripe orientation with a period Λ of around 1000 nm is noticed for all pattern sizes.

We also investigated freestanding patterns for the occurrence of single stripe orientation. Figure 5 illustrates square and circular disk‐shaped patterns of different sizes. Each pattern is connected to constrained film regions on two opposite sides by bridge‐like structures. In both shapes, the characteristic stripe microstructure is observed with a stripe period of around 1000 nm. Interestingly, a single stripe orientation is observed in both shapes and across all sizes (from 20 to 100 µm). This observation reveals that a transition to a multi‐stripe state likely occurs at larger feature sizes. Indeed, we would like to point out that the multi‐stripe state observed in the 20 µm wide freestanding bridge (Figure 3a) is a rare occurrence in our study, and most of the freestanding patterns exhibit a single stripe state below 100 µm pattern size.

When comparing freestanding and constrained patterns, we observe a fundamentally different correlation of pattern size and macroscopic boundaries. In the constrained condition, single colony patterns occur due to probability and not finite size‐effects. We attribute this observation to the constrained interface to the substrate, which hinders the re‐arrangement of mesoscopic twin boundaries required to reach the thermodynamic ground state of no macroscopic boundary. Instead, transformation kinetics decides on the martensitic microstructure at the length scale of mesoscopic twin colonies. The kinetic argument was recently confirmed by experiments with fast cooling rates, which have a strong influence on the colony size [36]. From the analysis of continuous films [18, 30], macroscopic boundaries originate during the growth of differently oriented martensite nuclei. The nucleation sites are likely not influenced by the patterning process, and thus, the number distribution of colonies is not affected. While nucleation starts preferentially from the film surface [30], our experiments do not show any influence of pattern edges on the colony statistics. In freestanding patterns, we observe a single stripe orientation in all pattern sizes, and hence we predict that the single stripe region exceeds the 100 µm range. In contrast to constrained patterns, re‐arrangement of twin boundaries is feasible in freestanding patterns, and thus the martensite microstructure can approach its thermodynamic ground state. However, the invariant line constraint results in a unique microstructure arrangement in freestanding films [15]. The nucleation and growth model of constrained films is no longer appropriate here, and it is not yet clear if the nucleation of a certain stripe orientation conditions the orientation of neighboring stripe nuclei over several tens of micrometers, as observed from experiments.

### 2.3 Scaling Effects with Film Thickness

Materials with uniaxial magnetocrystalline anisotropy and easy‐axis of magnetization aligned out‐of‐plane often exhibit a magnetic microstructure with band or stripe‐like appearance [10]. This magnetic microstructure results from minimizing the sum of magnetostatic and domain wall energies, leading to an equilibrium domain width. Kittel proposed that the domain width scales as the square root of the sample dimension aligned with the easy‐axis [9]. This scaling relation for magnetic domains has been observed in Ni‐Mn‐Ga films when the film thickness is reduced [37]. Also, for the martensite microstructure of these constrained films, a corresponding scaling relation of mesoscopic twin boundary period with film thickness has been reported [38], which in this case minimizes the sum of elastic energy and twin boundary energy.

**FIGURE 6:**
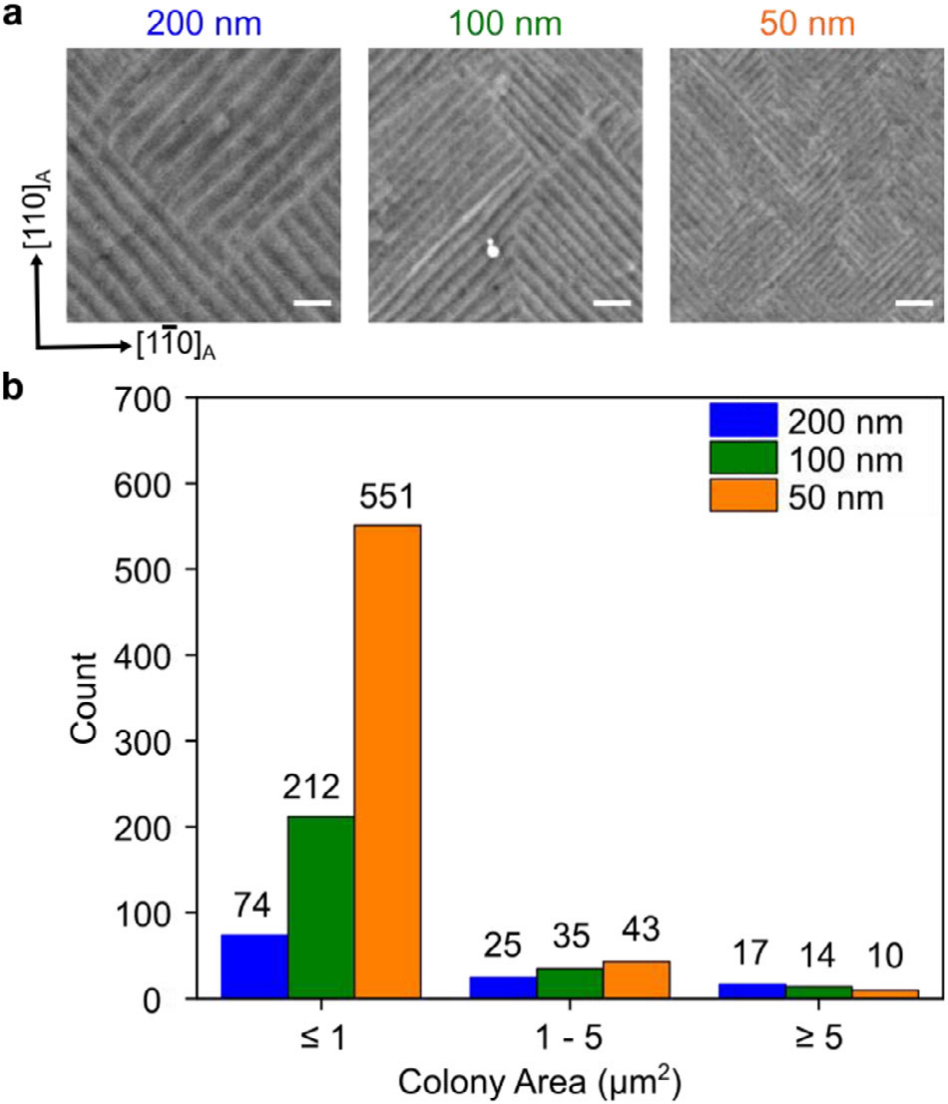
Effect of film thickness on the continuous constrained film martensite microstructure. (a) Secondary electron images depicting the martensite microstructure of 200 nm, 100 nm, and 50 nm thick Ni_52_Mn_27_Ga_21_ films grown on MgO(001) substrate. The white scalebar denotes 100 nm. (b) Bar chart summarizing the colony size distribution observed in these three film thicknesses after analyzing a film area of about 250 µm^2^ for each film.

For constrained films we revisited this experiment here by analyzing the martensite microstructure of 200 nm, 100 nm, and 50 nm thick continuous Ni‐Mn‐Ga films grown on MgO(001) substrate. Figure 6a depicts the observed martensite microstructure in these films. The films follow the same epitaxial growth relation on MgO(001) as on SrTiO_3_(001) and exhibit a hierarchical martensite microstructure. The mesoscopic twin boundary period analysis in these films reaffirms that it scales as the square root of the film thickness (see Figure S3). From the microstructure images, it is also apparent that the mesoscopic twin colony size reduces with the film thickness. We analyzed a film area of about 250 µm^2^ for each film thickness to obtain the distribution of colony size. The distribution in the three films is depicted in Figure 6b. As the film thickness is reduced from 200 nm to 50 nm, we notice a significant jump in the number of small‐sized colonies (area less than 1 µm^2^) and a decrease in the number of large‐sized colonies (area larger than 5 µm^2^).

Considering the nucleation scenario described in Section 2.2, the observations indicate that more primary nuclei form in thinner films. This observation can be rationalized by considering the martensitic transformation scenario in constrained thin films. The constraint imposed by a rigid substrate is known to impede the martensitic transformation of films, resulting in a lower martensitic transformation temperature with decreasing film thickness [39]. In other words, thinner films require higher undercooling during their transformation compared to a bulk crystal with the same composition. This promotes growth of more primary martensite nuclei and, accordingly, higher mesoscopic twin colony density with reducing film thickness.

**FIGURE 7:**
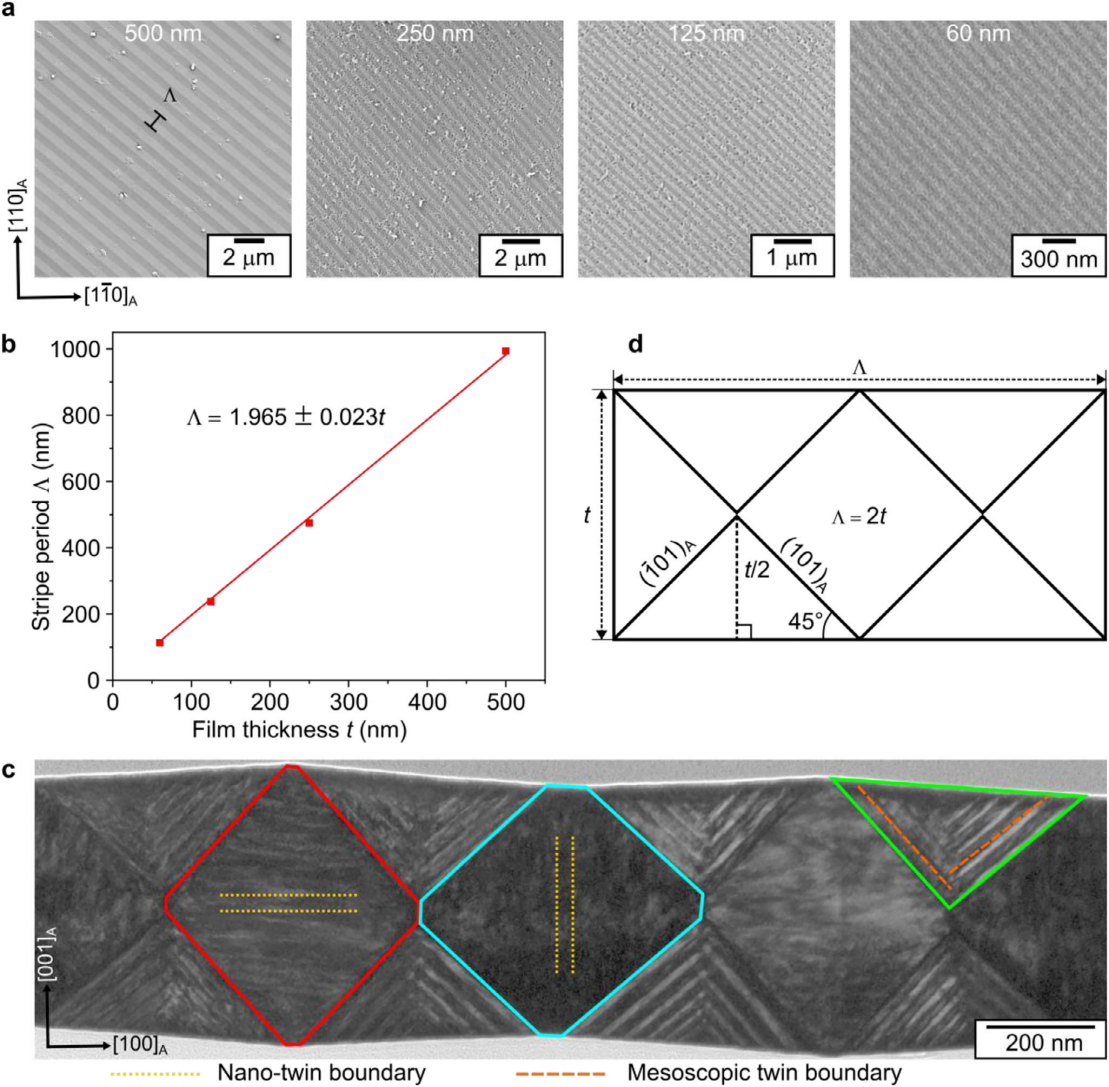
Microstructure evolution with film thickness in freestanding films. (a) Secondary electron images of 500, 250, 125, and 60 nm thick freestanding Ni‐Mn‐Ga‐Cu films depicting the evolution of martensite microstructure with film thickness. The variation in the stripe period is quite remarkable. (b) Plot depicting the relation between the stripe period in martensite microstructure and film thickness. The linear fit (solid red line) reveals stripe period is almost twice the value of the corresponding film thickness. (c) Bright‐field TEM cross‐section image of 500 nm‐thick Ni_52_Mn_18_Ga_25_Cu_5_ film across the stripes. The microstructure consists of a periodic arrangement of triangular domains (outlined in green) connecting thick and thin rhombus domains (outlined in red and blue). The triangular domains exhibit mesoscopic twin boundaries, while rhombus domains exhibit nano‐twin boundaries. (d) A simplified sketch of the cross‐section microstructure allows to derive the stripe period – film thickness relation using the geometry of triangular domains.

To understand the influence of film thickness on the martensite microstructure in freestanding condition, a series of Ni‐Mn‐Ga‐Cu films with film thicknesses ranging from 500 to 60 nm were grown on SrTiO_3_ buffered SOI substrates and subsequently released by underetching the top Si layer of the substrate. Figure 7a shows the martensite microstructure observed in the fabricated freestanding films. A parallel stripe microstructure is consistently observed, resulting from the invariant line constraint along the stripes [15]. No fundamental differences in the martensite microstructure occur with a reduction of film thickness, apart from the stripe period Λ, which decreases as the film thickness is reduced. To establish a correlation, the average stripe period of each film is plotted against film thickness in Figure 7b. The linear fit of the data reveals that the stripe period is nearly twice the film thickness. To understand the origin of this correlation, it is helpful to examine the film cross‐section microstructure shown in Figure 7c. The cross‐section of a 500 nm thick freestanding Ni_52_Mn_18_Ga_25_Cu_5_ film, prepared across the stripes, reveals a periodic arrangement of triangular and rhombus‐shaped domains containing twin boundaries (see Figure S4). As investigated in detail in a previous work [15], the absence of substrate constraint and finite thickness of the film result in this unique microstructure guided by invariant line constraint. It exhibits periodic variations in film thickness while preserving the lateral film extension [15]. The stripe appearance on the film surface originates from the thickness variations and the slightly tilted triangular domains observed in the film cross‐section. The scaling relation between Λ and film thickness can be derived from a simplified sketch of the cross‐section shown in Figure 7d. The triangular domains are bound by (101)_A_ and ($\bar{1}$01)_A_ mesoscopic twin planes that make 45° with the film surface. Using trigonometry, it is therefore evident that the stripe period equals twice the film thickness.

## 3 Summary and Outlook

In this work, we have investigated size‐effects in the ferroelastic martensite microstructure under constrained and freestanding conditions using epitaxially grown Ni‐Mn‐Ga‐based films as a model system. Based on the findings, we identify similarities and differences between ferroelastic and ferromagnetic patterns, which strongly depend if the ferroelastic pattern is constrained or freestanding:

- Pattern edges and their orientation do not influence the martensite microstructure of constrained and freestanding patterns. We attribute this finding to the well‐defined crystallographic orientations of twin boundaries, which does not allow for deviations in orientation when approaching pattern edges.
- A single colony pattern is the ferroelastic counterpart of a single ferromagnetic domain particle. Our analysis of constrained patterns reveals that this is just a probabilistic effect and not a physical finite size‐effect. For freestanding patterns, however, the size for single stripe orientation exceeds the examined pattern size of up to 100 µm.
- Film thickness, which is the shortest of the three dimensions in this study, emerges as the most important size parameter influencing the martensite microstructure. For constrained ferroelastic films a square‐root scaling law of mesoscopic twin boundary period with film thickness is observed, originating from a minimization of elastic and twin boundary energy. In contrast, we observe a linear scaling of stripe period with film thickness for freestanding ferroelastic films. The absence of substrate constraint allows to derive the linear relation using a geometrical model.

Our analysis focused on mesoscopic and macroscopic boundaries within the hierarchically twinned martensitic microstructure using pattern sizes in the micrometer range, relevant for microsystems [40]. As an outlook, it would be worthwhile to examine the influence of finite size on nanotwinning and martensitic transformation. Indeed, very recently constrained patterns down to 70 nm could be prepared. It allowed examining patterns with high aspect ratios, which showed a strong influence on the transformation temperatures [41]. This example illustrates that new effects at very small sizes still remain to be examined and explained.

## 4 Experimental

### 4.1 Film Deposition

Cu‐doped Ni‐Mn‐Ga films of various film thicknesses were epitaxially grown on commercially available 4 nm SrTiO_3_ buffered Si and Silicon‐On‐Insulator (SOI) substrates from Lumiphase AG by DC Magnetron sputtering, as described in our previous work [27]. Films of different thicknesses were prepared by co‐sputtering a Ni_48_Mn_22_Ga_30_ alloy target and a Cu elemental target on the substrates heated to 673 K. Epitaxial Ni‐Mn‐Ga films of various film thicknesses were also grown on single crystal MgO (001) substrates heated to 673 K by sputtering a Ni_48_Mn_32_Ga_20_ target.

### 4.2 Film Microfabrication

The fabrication of constrained Ni‐Mn‐Ga‐Cu patterns was performed by laser photolithography and Ar ion beam etching of a 500 nm‐thick film grown on Si substrate [26]. A 1.5 µm‐thick AZ 15nXT photoresist, used as mask, was exposed using an MLA 100 laser writer with a 365 nm wavelength and then developed in AZ 726 MIF solution. The Ar ion beam etching of the film was performed using a Scia Mill 150 tool, with Ar gas flow rate of 12 sccm in an ion source, He substrate back‐cooling flow rate of 5 sccm, a beam voltage of 700 V, sample stage tilts of 3° and 60°, and sample stage cooling chiller temperature at 273 K. To achieve freestanding Ni‐Mn‐Ga‐Cu patterns and films, the films deposited on SOI substrates were similarly microfabricated, and the top Si layer of SOI substrate was selectively under‐etched using a Xactix e2 XeF_2_ gas etching system with a chamber pressure of 400 Pa and appropriate etch durations based on the pattern size.

### 4.3 Film Characterization

The crystal structure of the 500 nm continuous Ni_52_Mn_19_Ga_25_Cu_4_ film grown on SrTiO_3_ (001) buffered Si and 200 nm thick Ni_52_Mn_27_Ga_21_ film grown on MgO (001) substrates were characterized using a Bruker AXS D8 Advance X‐ray diffractometer with Co radiation (*λ*_Κα_ = 0.178897 nm). A *θ ‒ 2θ* scan in Bragg‐Brentano geometry with sample tilt offsets (Δ*ω*) from 0° to 10° (step size = 1°) was used to account for the slight tilt in the crystal planes of martensite [42]. The films contain 14M martensite and NM (non‐modulated) martensite phases at room temperature (see Figure S5). The film microstructure and composition were analyzed using a ZEISS Sigma 300 scanning electron microscope fitted with a SmartEDX detector and a ThermoFisher Scientific Apreo 2S scanning electron microscope fitted with Ultim Max EDX detector from Oxford Instruments. Using a bulk Ni_2_MnGa standard, the composition of the investigated films was determined with 1 at.% accuracy. The composition of the investigated films is summarized in Table S1. The in situ microstructure analysis of a partly freestanding pattern during a martensitic transformation cycle was performed in a ZEISS Leo Gemini 1530 scanning electron microscope using a Kammrath & Weiss heating stage with temperature control (accuracy = 1 K). An FEI Quanta 3D FEG device and FEI Helios Nanolab 600i were used for preparing film cross‐section lamellae by focused ion beam (FIB) technique. The TEM investigation of the lamellae was done in FEI Talos F200 and JEM‐2200FS transmission electron microscopes operated at 200 kV. All crystallographic directions indicated in the Results and Discussion Section are based on L2_1_ convention indicated by ‘A’ subscript [43].

The magnetization behavior of 500 nm thick Ni_52_Mn_19_Ga_25_Cu_4_ film grown on SrTiO_3_ (001) buffered Si and 200 nm thick Ni_52_Mn_27_Ga_21_ film grown on MgO (001) were investigated by performing iso‐field measurements in a Quantum Design ‐VERSALAB vibrating sample magnetometer using an in‐plane applied magnetic field of 0.1 T along [110]_A_ direction and scan range of 400–150 K at a rate of 0.033 K s^−1^. The films are ferromagnetic at 300 K (see Figure S6).

## Conflicts of Interest

The authors declare no conflicts of interest.

## Supporting information




**Supporting File 1**: smll72699‐sup‐0001‐SuppMat.docx.

**Supporting File 2**: smll72699‐sup‐0002‐Video S1.mp4.

## Data Availability

The data that support the findings of this study are openly available in [RODARE] at [https://doi.org/10.14278/rodare.4016], reference number [4016].
