# Supplementary material for: Finite Size‐Effects in Martensite Microstructure of Magnetic Shape Memory Films
Source: Small. 2026 Feb 4;22(19):e12162. doi: 10.1002/smll.202512162 (PMC13040122; doi:10.1002/smll.202512162)
Supplement: Supplementary file 1 — Supporting File 1: smll72699‐sup‐0001‐SuppMat.docx. [file SMLL-22-e12162-s001.pdf]

Supporting Information

Finite Size-Effects in Martensite Microstructure of Magnetic Shape Memory Films

Satyakam Kar[1,2,3], *Aman Singh*[2,4], *Kornelius Nielsch*[1,2], *Heiko Reith*[1], *and Sebastian Fähler*[2,3,*]

[1]Leibniz IFW Dresden, Institute for Metallic Materials, 01069 Dresden, Germany

[2]TU Dresden, Institute of Materials Science and Institute of Applied Physics, 01062 Dresden, Germany

[3]Helmholtz-Zentrum Dresden-Rossendorf, 01328 Dresden, Germany

[4]Leibniz IFW Dresden, Institute for Emerging Electronic Technologies, 01069 Dresden, Germany

*Corresponding author, e-mail: s.faehler@hzdr.de , Tel.: +493512602775, Postal address: Helmholtz-Zentrum Dresden-Rossendorf, Bautzner Landstraße 400, 01328 Dresden, Germany

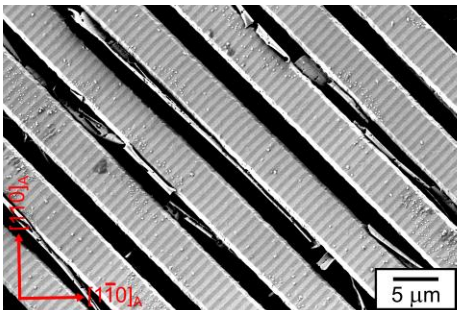

Figure S1. Secondary electron image depicting the martensite microstructure of 4 µm-wide $Ni_{52}Mn_{19}Ga_{25}Cu_4$ freestanding bridges. The stripe orientation and period are identical to wider bridge structures depicted in Figure 3b and 3c of the paper.

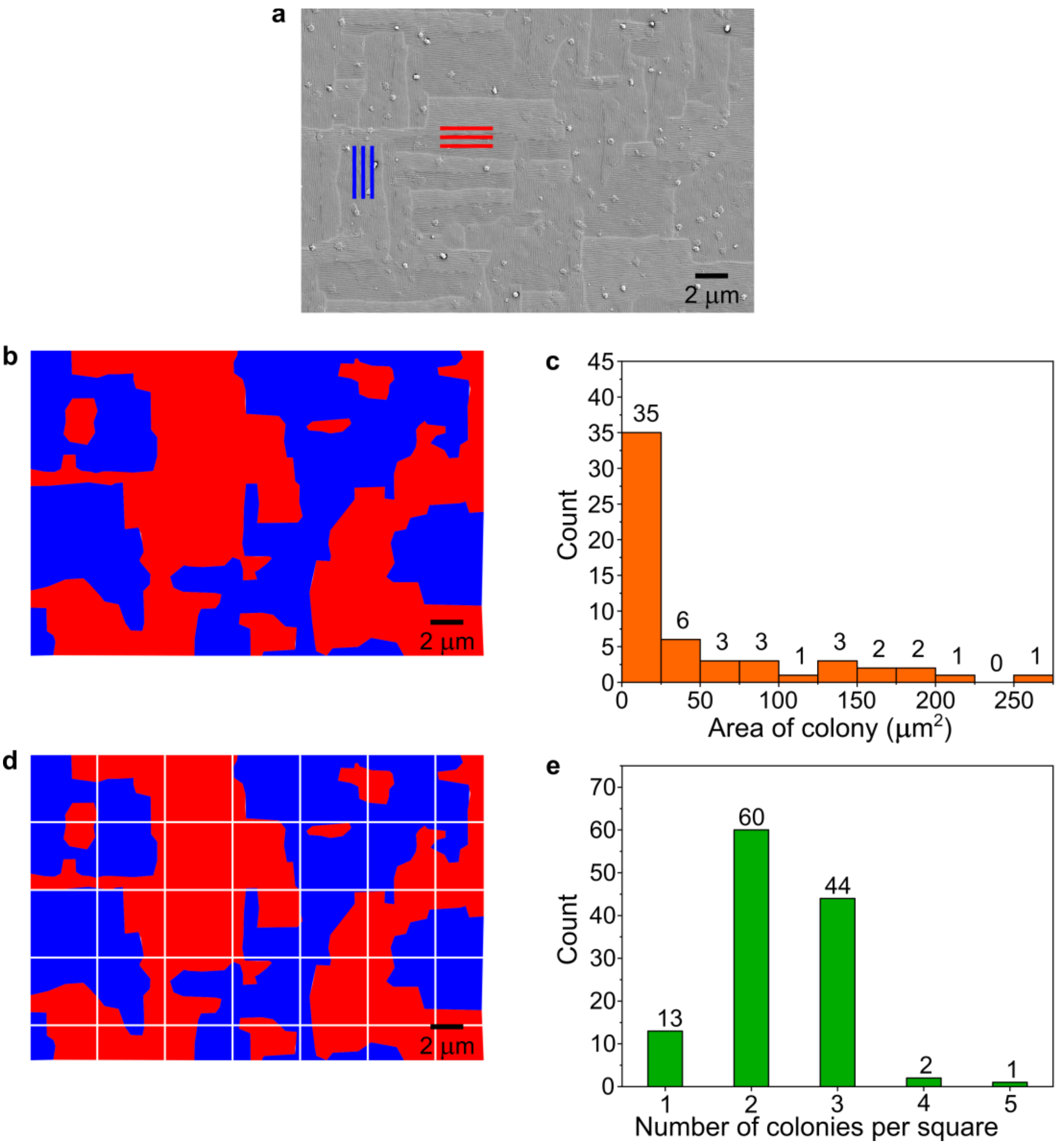

Figure S2. Martensite microstructure analysis of continuous constrained 500 nm thick $Ni_{52}Mn_{19}Ga_{25}Cu_4$ film as a reference. (a) The martensite microstructure exhibits two trace directions of mesoscopic twin boundaries highlighted in red and blue. (b) The regions with a single trace direction are grouped as a 'colony.' These colonies are now colored as per their trace direction. (c) Histogram depicting the colony distribution obtained after analyzing five images, each with an area of 511 µm^2. (d) The analysis is then repeated by segmenting the images into 4 µm squares (24 squares per image) and calculating the number of colonies in each square. (e) Bar chart depicting the distribution of colonies obtained from the segmented squares.

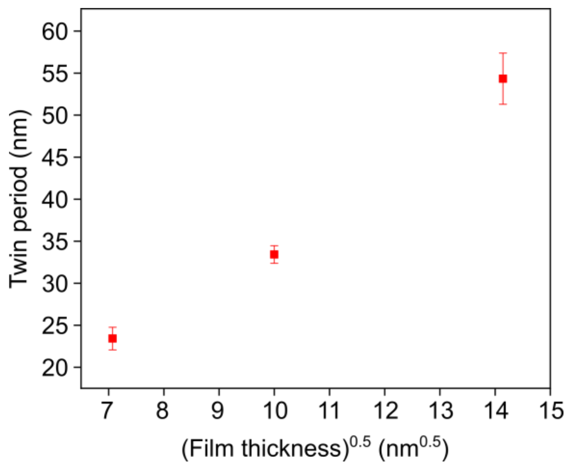

Figure S3. Plot depicting the correlation between mesoscopic twin boundary period and film thickness in constrained epitaxial Ni-Mn-Ga films grown on MgO (001) substrate.

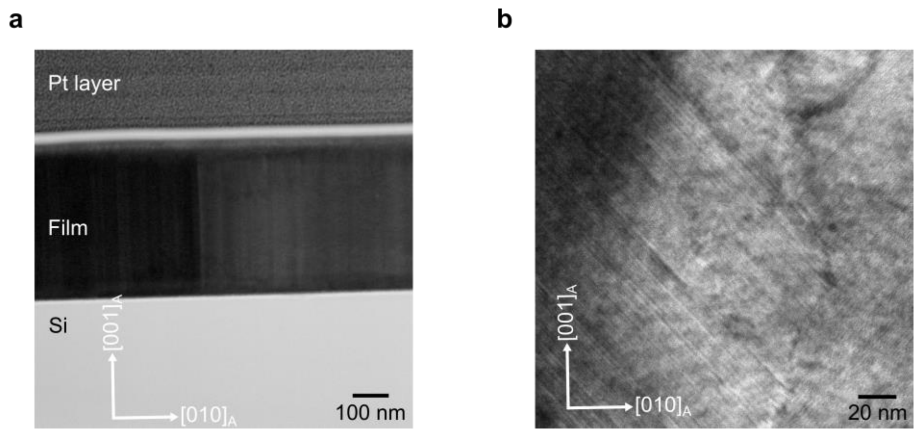

Figure S4. Bright field STEM images depicting the microstructure of (a) thin rhombus domain and (b) thick rhombus domain as it appears in the freestanding film cross-section prepared along the stripes. The {110} nanotwin boundaries appear parallel to film thickness in the thin rhombus domain and tilted at 45° in the thick rhombus domain.

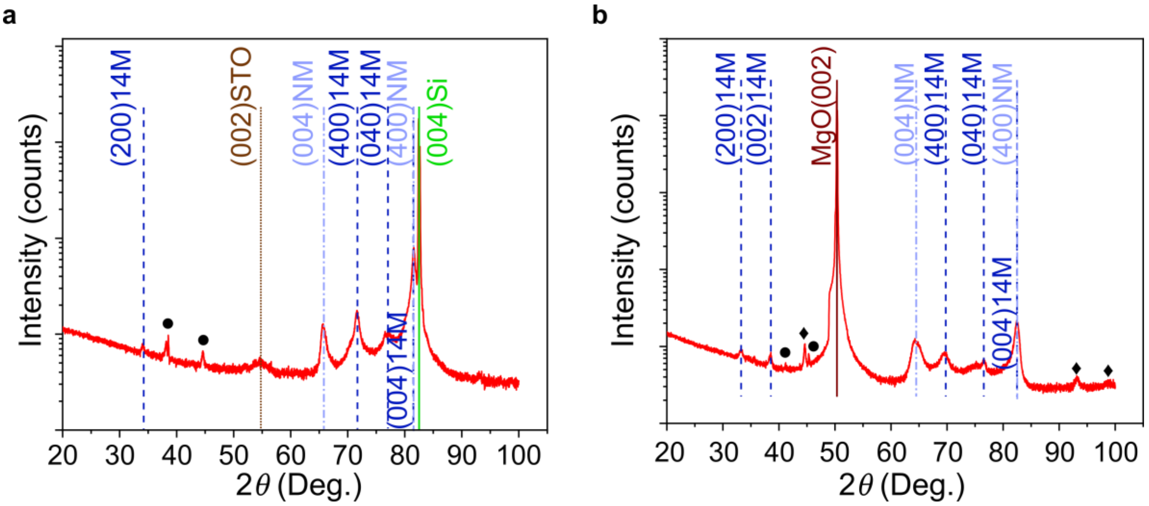

Figure S5. XRD diffractogram of (a) 500 nm-thick $Ni_{52}Mn_{19}Ga_{25}Cu_4$ film grown on $SrTiO_3$ (STO)(001) buffered Si substrate and (b) 200 nm thick $Ni_{52}Mn_{27}Ga_{21}$ film grown on MgO(001) substrate. Peaks indicated by black dots originate from the sample holder. The peaks indicated by black diamond originate from silver paste used for conduction in the prior electron microscopy investigation of the sample. Both films have 14M and NM martensite phases at room temperature.

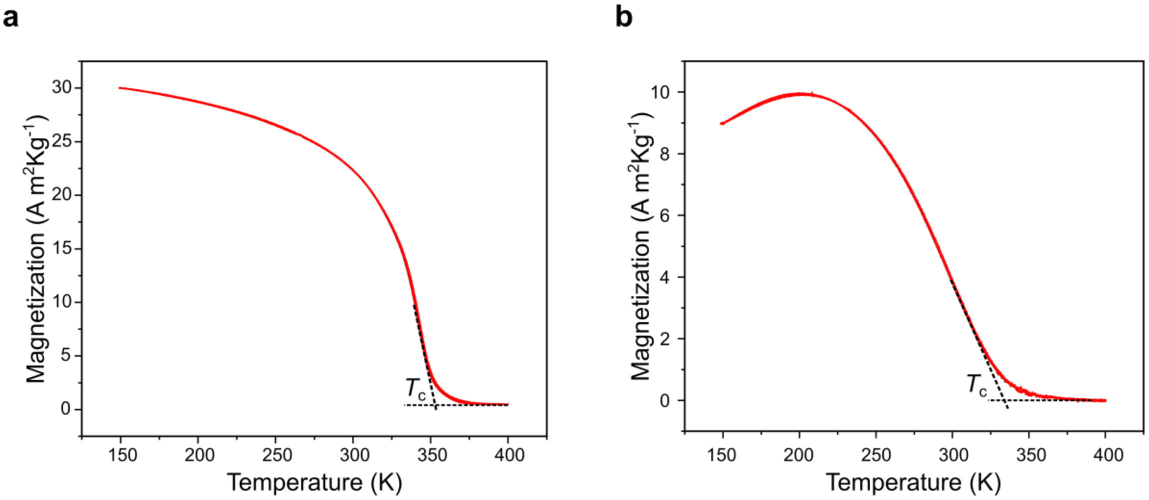

Figure S6. *M* vs. *T* plots of (a) 500 nm-thick $Ni_{52}Mn_{19}Ga_{25}Cu_4$ film grown on $SrTiO_3$ (STO)(001) buffered Si substrate and (b) 200 nm thick $Ni_{52}Mn_{27}Ga_{21}$ film grown on MgO(001) substrate measured at an applied magnetic field of 0.1 T. The films are ferromagnetic at 300 K, with Curie temperature T_c at 352 K (500 nm film) and 337 K (200 nm film). No first order transition corresponding to martensitic transformation is observed in both films, indicating that the transformation occurs above the T_c.

Table S1. Overview of film compositions of the investigated Ni-Mn-Ga-based films with their reference in the manuscript.

**Manuscript reference**	**EDX Film composition**
Figure 1, 2, 3, 4, and 5	$Ni_{52}Mn_{19}Ga_{25}Cu_4$
Figure 6a	$Ni_{52}Mn_{27}Ga_{21}$
Figure 7a- 500 nm	$Ni_{52}Mn_{18}Ga_{25}Cu_5$
Figure 7a- 250 nm	$Ni_{52}Mn_{17}Ga_{27}Cu_4$
Figure 7a- 125 nm	$Ni_{51}Mn_{17}Ga_{28}Cu_4$
Figure 7a- 60 nm	$Ni_{50}Mn_{17}Ga_{28}Cu_5$
